# MET signalling in primary colon epithelial cells leads to increased transformation irrespective of aberrant Wnt signalling

**DOI:** 10.1038/sj.bjc.6602405

**Published:** 2005-03-22

**Authors:** E M J Boon, M Kovarikova, P W B Derksen, R van der Neut

**Affiliations:** 1 Academic Medical Centre, Department of Pathology, University of Amsterdam, Meibergdreef 9, 1105 AZ Amsterdam, The Netherlands; 2 Institute of Biophysics, Acad. Sci. Czech Rep., Kralovopolska 135, CZ – 612 65 Brno, Czech Republic

**Keywords:** MET, colon, HGF, transformation

## Abstract

It has been shown that in hereditary and most sporadic colon tumours, components of the Wnt pathway are mutated. The Wnt target MET has been implicated in the development of colon cancer. Here, we show that overexpression of wild-type or a constitutively activated form of MET in colon epithelial cells leads to increased transformation irrespective of Wnt signalling. Fetal human colon epithelial cells without aberrant Wnt signalling were transfected with wild-type or mutated MET constructs. Expression of these constructs leads to increased phosphorylation of MET and its downstream targets PKB and MAPK. Upon stimulation with HGF, the expression of E-cadherin is downregulated in wild-type MET-transfected cells, whereas cells expressing mutated MET show low E-cadherin levels independent of stimulation with ligand. This implies a higher migratory propensity of these cells. Furthermore, fetal human colon epithelial cells expressing the mutated form of MET have colony-forming capacity in soft agar, while cells expressing wild-type MET show an intermediate phenotype. Subcutaneous injection of mutated MET-transfected cells in nude mice leads to the formation of tumours within 12 days in all mice injected. At this time point, mock-transfected cells do not form tumours, while wild-type MET-transfected cells form subcutaneous tumours in one out of five mice. We thus show that MET signalling can lead to increased transformation of colon epithelial cells independent of Wnt signalling and in this way could play an essential role in the onset and progression of colorectal cancer.

Colorectal cancer (CRC) is common in the Western world and accounts for a high amount of cancer-related deaths. Advances in molecular genetics have greatly increased our understanding of the development of CRC (Liu *et al*, 1992; Kinzler and Vogelstein, 1996; Korinek *et al*, 1997). Colorectal cancer evolves through a series of morphologically recognisable stages known as the adenoma–carcinoma sequence (Vogelstein *et al*, 1988). Mutations involving components of the Wnt signalling pathway are essential for the initial neoplastic transformation. These mutations lead to accumulation of *β*-catenin in the nucleus, where it activates TCF-mediated transcription (Bienz and Clevers, 2000). Transcription of TCF-regulated genes, including cyclinD1 (Shtutman *et al*, 1999), c-Myc (He *et al*, 1998) and CD44 (Wielenga *et al*, 1999), is thought to impose a stem cell phenotype on colon cells and provides a basis for their proliferative capacity. Recently, we identified the receptor tyrosine kinase MET as a target of Wnt signalling, suggesting a role for MET in controlling the turnover and differentiation of intestinal epithelium (Boon *et al*, 2002).

Apart from being crucial in mammalian development and epithelial morphogenesis, MET is overexpressed in several human cancers and germline mutations of the *MET* gene have been reported in hereditary papillary renal carcinoma (Schmidt *et al*, 1997). These mutations induce enhanced enzymatic activity of the MET tyrosine kinase, which may lead to increased transforming and tumorigenic properties (Jeffers *et al*, 1997). Activation of MET by either ligand stimulation or overexpression of the receptor is thought to depend upon receptor dimerisation or oligomerisation, resulting in transphosphorylation. In addition, different mutations in the tyrosine kinase domain have been found, including L1213V and M1268T, which lead to constitutive activation of the receptor (Jeffers *et al*, 1997; Schmidt *et al*, 1997; Jeffers *et al*, 1998).

In CRC, MET is overexpressed in the vast majority of adenomas, invasive carcinomas, and metastases (Di Renzo *et al*, 1991, 1995; Liu *et al*, 1992; Wielenga *et al*, 2000). In addition, MET has been shown to be amplified in primary colorectal tumours as well as in liver metastasis (Di Renzo *et al*, 1995). We previously showed that MET overexpression in the initial phase of CRC is ‘regulatory’ and results from enhanced Wnt signalling (Boon *et al*, 2002). We furthermore hypothesise that in addition to Wnt signaling, other pathways could regulate MET expression and that overexpression of MET could also have an effect on tumorigenesis irrespective of Wnt signalling.

In the present study, we show that overexpression of wild-type MET or a constitutively active form of MET in primary fetal human colon epithelial cells leads to an initial growth advantage, irrespective of aberrant Wnt signalling, resulting in increased tumorigenicity.

## MATERIALS AND METHODS

### Plasmid constructions

All constructs described were subcloned into pcDNA3.1. To generate the expression vector, a 4.2 kb *Bam*HI–*Bam*HI fragment of mouse Met (kindly provided by George Vande Woude) was subcloned into the *Bam*HI sites of pcDNA3.1 vector. The resulting plasmid was designated wild-type Met (WTMet). Site-directed mutagenesis was used to generate the mutations L1213V and M1268T in Met, and mutations were verified by sequencing both strands of DNA. This plasmid was designated Met mutated (Metmut). As a control, the empty vector was used.

### Cell culture and transfections

Primary fetal human colon epithelial cells (FHC) (CRL-1831, ATCC, Manassas, VA, USA) were cultured in a 1 : 1 mixture of DMEM and Ham's nutrient mixture, supplemented with 10% fetal calf serum (FCS), 25 mM HEPES, 10 ng ml^−1^ cholera toxin, 5 *μ*g ml^−1^ insulin, 5 *μ*g ml^−1^ transferrin and 100 ng ml^−1^ hydrocortisone. DLD1 (kindly provided by H Clevers) and HCA-7 cells (parental cell line kindly provided by J Tuynman) were grown in RPMI medium and DMEM medium, respectively, supplemented with 10% FCS, 100 U ml^−1^ penicillin and 100 *μ*g ml^−1^ streptomycin (all from Life Technologies, Paisley, UK). Cells were transiently transfected with either 5 *μ*g empty vector, WTMet or Metmut plasmids using lipofectamine (Invitrogen, Paisley, UK). To correct for differences in transfection efficiency, cells were cotransfected with a reporter plasmid containing a GFP gene (Clontech., Palo Alto, CA, USA). The ratio of Met-containing plasmid to GFP-encoding plasmid was 5 : 1. Transfection efficiency was determined in live cells by counting, with a fluorescence microscope fitted with phase contrast optics, the number of GFP-positive cells as well as the total cell number in 10 independent optical fields. The resulting transfection efficiency was used to correct for differences in transfection in each culture well in each experiment.

### TCF reporter assay

Cells were transiently transfected with either pTOPflash or pFOPflash reporter plasmids (Upstate Biotechnology, Lake Placid, NY, USA) and different Met-encoding plasmids using lipofectamine (Invitrogen, Paisley, UK). To correct for differences in transfection efficiency, cells were cotransfected with a Renilla luciferase encoding plasmid. All experiments were performed in triplicate. At 48 h after transfection, cells were lysed in luciferase reporter lysis buffer and monitored for luciferase and renilla activity using a Dual-Luciferase Reporter Assay System (Promega, Madison, NY USA). Light units were recorded using a luminometer.

### Western Blot analysis

Cells were transiently transfected using lipofectamine. At 30 h after transfection, cells were starved overnight in medium containing 0.1% serum, after which cells were stimulated with 100 ng ml^−1^ HGF for 3 min (Relia Tech. Inc., Breda, The Netherlands). At 48 h after transfection, protein extracts were prepared in lysis buffer (10 mM Tris pH 8.0; 15 mM NaCl; 1% NP40, 10% glycerol; 0.4 mg ml^−1^ sodium orthovanadate). Proteins were separated by SDS–PAGE and blotted onto immobilon-P transfer membranes (Millipore Corp., Bedford, MA, USA) by tank blotting. Membranes were blocked in Tris-buffered saline (100 mM Tris-Cl pH 7.5; 150 mM NaCl) containing 0.1% Tween (Sigma, St Louis, MO, USA) and 5% nonfat dry milk, probed with monoclonal antibodies – AC-15 (against *β*-Actin) (Sigma), HECD-1 (against E-cadherin) (Takara Bio Inc., Otsu, Shiga, Japan) – and polyclonal antibodies – Rabbit anti-Met SP260 (Santa Cruz Biotechnology, Santa Cruz, CA, USA), Rabbit anti-c-Met phosphospecific pYpYpY^1230/1234/1235^ (Biosource, Camarillo, CA, USA), rabbit anti-Phospho-Akt (ser 473) (Cell Signalling Technology, Beverly, MA, USA), rabbit anti-Akt (Santa Cruz Biotechnology, Santa Cruz, CA, USA), rabbit anti- MAPK (Erk 2) (Santa Cruz Biotechnology, Santa Cruz, CA, USA) or rabbit anti-Phospho-p44/42 MAPK(Thr 202/Tyr 204) (New England Biolabs, Hitcin, UK). Proteins were detected with horseradish peroxidase-conjugated secondary antibodies (Dakopatts, Glostrup, Denmark) in a standard chemiluminescence Western Blotting protocol (ECL Western Blotting, Amersham Pharmacia Biotech Inc., Aylesbury, UK).

### Confocal microscopy

Fetal human colon epithelial cells were grown to 60% confluency on glass coverslips in six-well plates. Cells were transiently transfected with either 5 *μ*g empty vector, WTMet or Metmut plasmids using lipofectamine. At 30 h after transfection, cells were starved overnight in medium containing 0.1% serum, after which cells were grown for 24 h with 100 ng ml^−1^ HGF. Cells grown on coverslips were fixed in cold methanol and processed for indirect immunofluorescence. Slides were stained with a mouse monoclonal antibody HECD-1 (Takara Bio Inc., Otsu, Shiga, Japan) against E-cadherin, and bound antibodies were detected using RPE-conjugated goat anti-mouse antibodies (Jackson Laboratory, Main, USA). Slides were analysed on a Leica TCS NT confocal microscope (Leica, Inc., Heidelberg, Germany).

### Soft agar assay

For analysis of colony formation in soft agar, cells were trypsinised 2 days after transfection, counted and 5000 cells were resuspended in complete medium containing 0.5% seaplaque agar (Bioproducts, Rockland, ME, USA). Cells were seeded in six-well plates (2 ml well^−1^) containing a 1% seaplaque agar underlay and supplemented three times a week with complete medium. After 10 days of growth, colonies were stained with an aqueous solution containing 0.5 mg ml^−1^ NADH with 0.5 mg ml^−1^ nitro blue tetrazolium salt. Following overnight incubation at 37°C, colonies were counted using Image Pro software.

### Tumorigenicity assay

Transfected cells (2.5 × 10^5^) were injected subcutaneously in the neck of 6-week-old male nude mice (BALB/cAnNCrl-nuBR, Charles River) (*n*=5 per experimental group). Mice were examined every 2 days and tumour length and width were measured using calipers. After 19 days, mice were euthanized and tumours, livers and spleens were excised for histopathological processing.

## RESULTS

### HCA-7 cells have high expression of MET protein levels independent of active Wnt signalling

**Figure 1:**
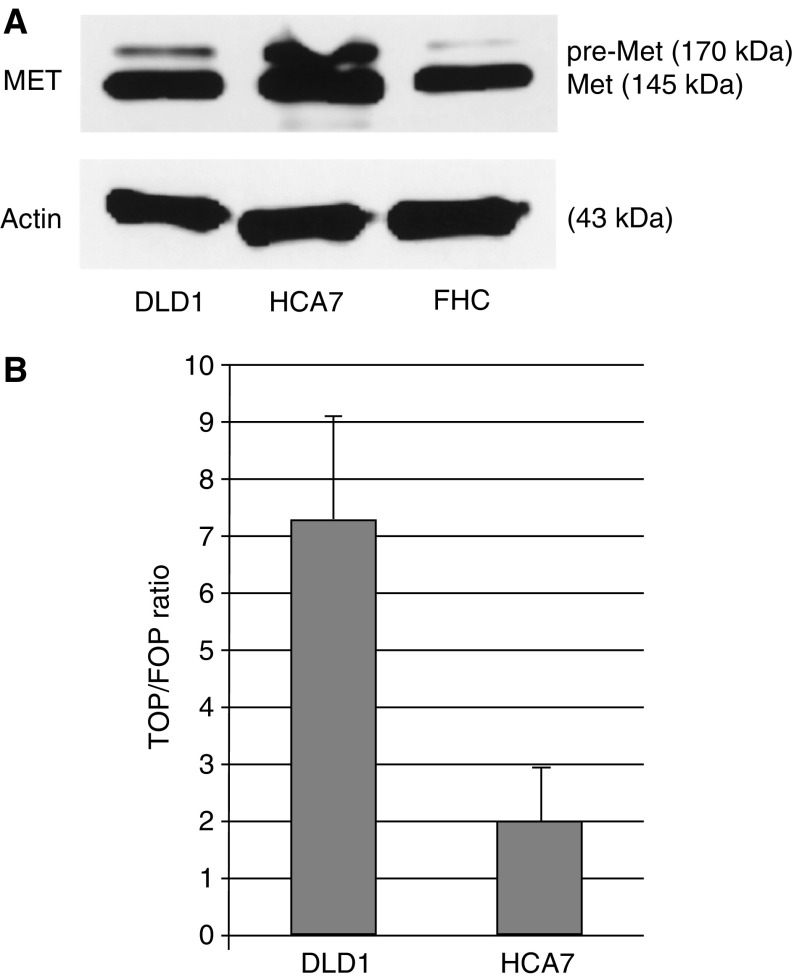
HCA-7 cells have high expression of MET protein levels independent of aberrant Wnt signalling. MET protein levels in HCA-7 cells were compared to the MET levels in a CRC cell line DLD1 and in fetal colon epithelial cells. As a loading control, *β*-Actin was used (**A**). This Met expression was independent of active Wnt signalling as shown by the TOP/FOP ratios present in these cells compared to DLD1 cells, containing active Wnt signalling (**B**).

In a human hereditary nonpolyposis colorectal cancer (HNPCC)-derived cell line (HCA-7), we observed a high level of MET expression. This MET protein expression was comparable to an established CRC cell line, DLD1, which contains mutations in the *APC* gene (Figure 1A). HCA-7 cells have, however, no mutations in APC or *β*-catenin (Ilyas *et al*, 1997; Rowan *et al*, 2000), resulting in low TCF-mediated transcription as shown in Figure 1B. The TOP/FOP ratio in DLD1 cells was 7.4 compared to TOP/FOP ratios below 2 in HCA7 cells (Figure 1B). High expression levels of Met without aberrant Wnt signalling in these cells indicates that expression of MET next to regulation by the Wnt pathway could be regulated by an additional signalling pathway. Furthermore, MET could have an effect on tumorigenesis irrespective of Wnt signalling.

### Fetal human colon epithelial cells have no aberrant Wnt signalling

**Figure 2:**
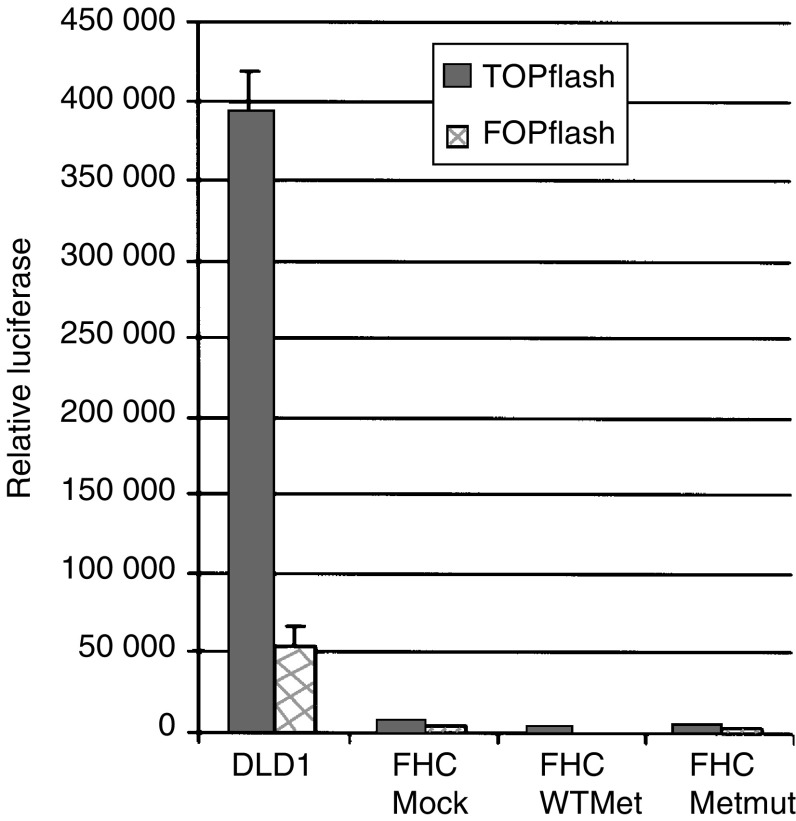
There is no active Wnt signalling in the fetal colon epithelial cells. TOPflash and FOPflash activities were measured in colon epithelial cells containing empty vector, WTMet or Metmut constructs. As a positive control for active Wnt signalling, TOPflash and FOPflash activities were also measured in DLD1 cells. Measurements were corrected for differences in transfection efficiency using a Renilla luciferase-encoded construct.

To explore our hypothesis that MET has transforming capacity irrespective of Wnt signalling, we used primary FHC epithelial cells. Using a TCF reporter assay, we found that these FHC epithelial cells have no detectable TCF-mediated transcription. We compared the TOPflash activity of the FHC cells to the TOPflash activity of DLD1 cells. The TOPflash activity of the colon epithelial cells transfected with empty vector or with different Met constructs was very low as compared to the DLD1 cells (Figure 2). The TOP/FOP ratio in DLD1 cells was 7.4 compared to TOP/FOP ratios below 2 in FHC cells. Therefore, no active Wnt signalling could be found in the FHC cells, irrespective of the overexpression of the wild-type or mutant Met.

### Expression of wild-type or mutated Met constructs in FHC epithelial cells leads to increased phosphorylation of Met and its downstream targets PKB and MAPK

**Figure 3:**
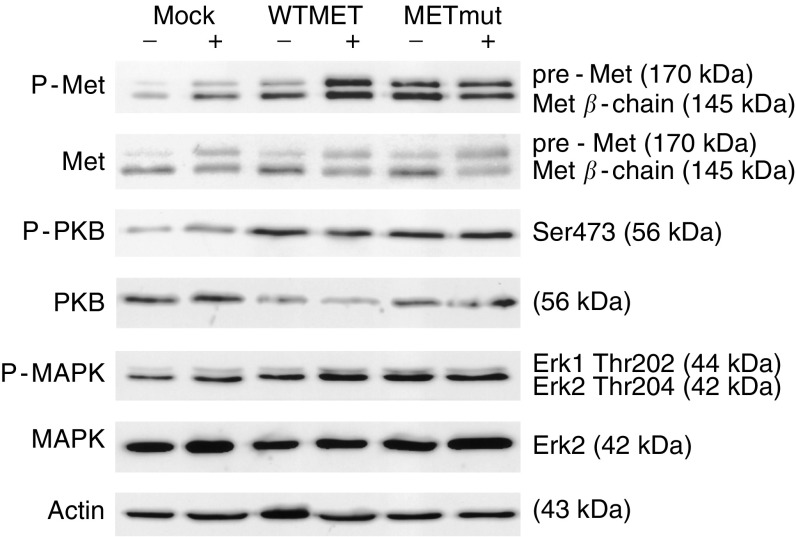
Overexpression of WT or mutated MET leads to higher phosphorylation levels of MET and its downstream targets PKB and MAPK. Fetal human colon epithelial cells were transiently transfected with empty vector, WTMet or Metmut constructs. Cells were stimulated with 100 ng ml^−1^ HGF for 3 min and protein lysates were prepared. Blots were stained with the following antibodies: anti-MET, anti-phospho-Met, anti-phospho-PKB, anti-phospho-MAPK and as loading control, anti-PKB, anti-MAPK and anti-*β*-Actin.

Although the colon epithelial cells have no active Wnt signalling, they have detectable Met protein levels. In addition, transient expression of wild-type or mutated Met constructs resulted in higher Met protein levels as compared to the mock-transfected cells containing endogenous Met protein (Figure 3). The increase in expression was relatively small due to a low transfection efficiency (10–20%). However, probing of the filter with anti-phospho Met antibody demonstrated that this level of overexpression of mutationally activated Met could lead to enhanced autophosphorylation levels of the receptor. This also gave rise to activation of the effector molecules PKB and MAPK, as shown by staining with anti-phospho PKB or anti-phospho MAPK antibodies, respectively (Figure 3). This effect was marked, although only 10–20% of the cells were transfected. As a control, total protein levels of PKB, MAPK and Actin were measured. Activation of Met and its downstream targets could still be increased by the addition of HGF in mock-transfected cells as well as in the cells transfected with WTMet constructs. However, mutations in the kinase domain of MET lead to constitutively activated Met signalling independent of ligand stimulation (Figure 3).

### E-cadherin levels are downregulated by the expression of MET constructs

**Figure 4:**
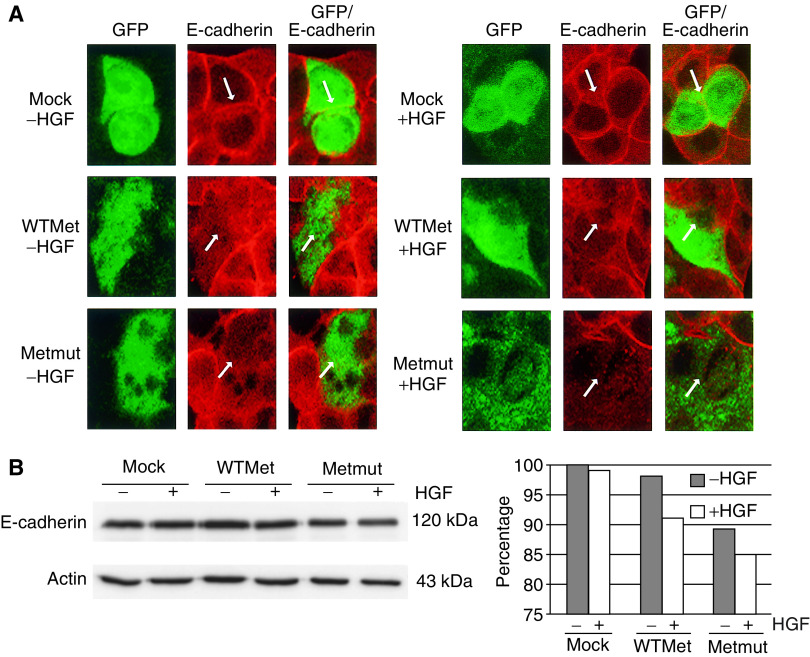
E-cadherin expression is downregulated by stimulation with HGF in mock- and WTMet-transfected cells or by the expression of constitutively active Met. Fetal human colon epithelial cells were cultured on glass coverslips and transfected with empty vector, WTMet or Metmut constructs and grown with or without the addition of 100 ng ml^−1^ HGF for 24 h. Slides were processed for indirect immunofluorescence and analysed on a confocal microscope for E-cadherin expression. Arrows depict cell–cell boundaries (**A**). In parallel, protein lysates were prepared and blots were stained for E-cadherin expression. As a control, *β*-Actin was used. The calculated values by densitrometry are shown for E-cadherin protein levels (**B**).

To translate these biochemical data to functional data, we looked at the scatter capacity of transfected colon epithelial cells. Since the transfection efficiency was limited in these cells (10–20% efficiency), it was important to distinguish between transfected and untransfected cells. In all transfection experiments, we therefore cotransfected the cells with a plasmid encoding GFP (at a ratio of 5 : 1 for the Met-containing plasmid : GFP-encoding plasmid) to control for transfection efficiency and for identification purposes. By observing the GFP-positive cells using confocal microscopy, we could identify the transfected cells. Expression levels of E-cadherin were thus investigated in these GFP-positive cells only. E-cadherin is a component of adherens junction complexes. Previous studies have shown that the level of E-cadherin is negatively correlated with the migratory/scatter capacity of colon carcinoma cells (Masur *et al*, 2001). Confocal microscopy analysis of transfected cells on glass coverslips revealed high levels of E-cadherin in mock-transfected cells and slightly diminished levels of E-cadherin after 24 h HGF stimulation (Figure 4A, upper panel). Cells containing the wild-type Met construct showed intermediate levels of E-cadherin, with reduced levels after ligand stimulation (Figure 4A, middle panel). Low levels of E-cadherin were observed in cells containing the mutated form of Met independent of ligand stimulation (Figure 4A, lower panel). In parallel, protein lysates of the transfected cells were prepared and E-cadherin levels were studied by Western blot analysis. Blots were analysed by densitometry and signals were normalised for loading by comparison with the appropriate *β*-actin signal. E-cadherin levels were downregulated up to 7% in WTMet-overexpressing cells (Figure 4B). In cells transfected with a mutant Met construct, E-cadherin levels were downregulated by 15% even without ligand stimulation (Figure 4B). Thus, even on a background of 80–90% untransfected cells, the downregulation of E-cadherin could still be visualised by Western blot analysis.

### Expression of WT or mutated Met constructs leads to colony formation of colon epithelial cells

**Figure 5:**
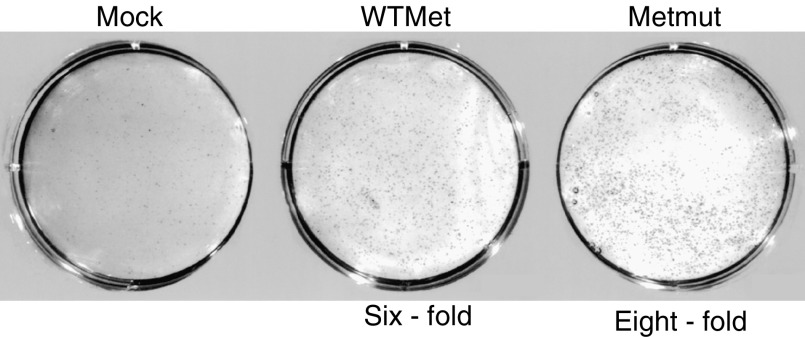
Overexpression of WT or mutated MET leads to colony formation in soft agar. Fetal human colon epithelial cells were transfected with empty vector, WTMet or Metmut constructs, trypsinised and 5000 cells were used in a soft agar assay. Cells were grown in soft agar for 10 days, after which colonies were stained and counted. Increase in the amount of colonies is determined in comparison to the amount of colonies from the mock-transfected cells.

To further explore our hypothesis that overexpression of MET, irrespective of Wnt signalling, leads to transformation of primary colon epithelial cells, we performed soft agar assays with the transfected cells. Colony formation of cells in soft agar is used as a method to identify the capacity to grow anchorage independent and is a measurement for transformed cells. This assay thus gives more information about growth, survival and transformation of cells. Mock-transfected cells did not grow efficiently in soft agar, while cells overexpressing wild-type or mutant Met proteins were able to form colonies in soft agar within 10 days (Figure 5). Expression of mutated Met resulted in high transforming capacity, leading to the largest number and size of colonies compared to mock- and wild-type Met-transfected cells (Figure 5). The increase in the amount of colonies compared to mock-transfected cells was six-fold for WTMet-transfected cells and mutant Met-transfected cells formed eight times more colonies in soft agar (average values from five independent experiments).

### Tumour formation in nude mice

**Figure 6:**
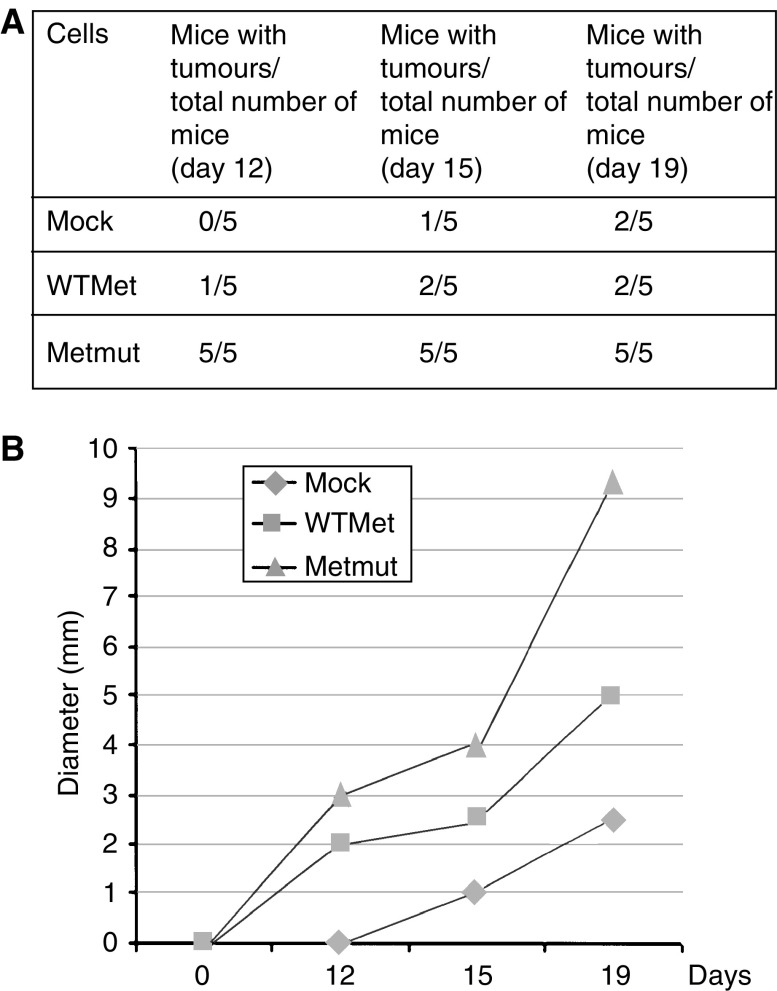
Overexpression of WT or mutated MET leads to tumour growth in nude mice. At 24 h after transfection with WTMet or Metmut constructs, FHC cells were injected into nude mice (five mice per group). Mice were monitored for tumour growth. First tumours were visible 12 days after injection and were followed for a total of 19 days. The number of mice with tumours (**A**) and tumour size (**B**) were measured in time.

To investigate whether these *in vitro* data have an *in vivo* correlate, we injected the transfected colon epithelial cells subcutaneously in the neck of nude mice. Within 12 days after injection, cells transfected with mutated Met formed tumours in all treated mice (five out of five mice). At this time point, only one out of five mice of the WTMet cell-injected group had a palpable tumour, but none of the mice injected with cells containing empty vector had tumours (Figure 6A). Tumour growth was followed for a total of 19 days. Cells containing a mutated form of the Met receptor had an initial growth advantage over WTMet- or mock-transfected cells (Figure 6A and B). No metastases were detected in the liver or the spleen after 19 days. Thus, overexpression of wild-type or constitutively active Met leads to transformation of colon epithelial cells independent of active Wnt signalling in both *in vitro* as well as *in vivo* models.

## DISCUSSION

Transformation of normal intestinal epithelial cells to dysplastic cells and eventually carcinoma cells comprises different stages (Vogelstein *et al*, 1988). Within these stages, several genes are up/downregulated or mutated, leading to tumour progression. It has been shown that the earliest neoplastic lesions in CRC are caused by mutations in components of the Wnt signalling pathway including APC and *β*-catenin (Bienz and Clevers, 2000). Also, in more than 85% of the sporadic tumours, the *APC* gene is mutated, indicating the importance of active Wnt signalling and subsequent transcription of TCF target genes in the onset of CRC. Our group has shown that the receptor tyrosine kinase MET is overexpressed during all stages of the adenoma–carcinoma sequence (Wielenga *et al*, 2000; Boon *et al*, 2002). Furthermore, we identified MET as a Wnt signalling target gene, indicating that the expression of Met in the majority of both hereditary as well as sporadic colon tumours is a consequence of active Wnt signalling (Boon *et al*, 2002). However, in a HNPCC-derived cell line (HCA-7), where constitutively active Wnt signalling is not present, we also observed high levels of MET expression. In addition to regulation by the Wnt pathway, expression of MET could therefore be regulated by other signalling pathways. This should be investigated in more detail. Nevertheless, it is evident that MET is overexpressed in colorectal tumours with or without the presence of active Wnt signalling, implying an important role for MET in the onset and progression of CRC. We, therefore, further investigated the role of MET on transformation of primary colon epithelial cells and explored the possibility that MET signalling could lead to an initial growth advantage independent of aberrant Wnt signalling, resulting in increased tumorigenicity.

To investigate the transforming capacity of the receptor tyrosine kinase MET in colon epithelium, we used FHC epithelial cells. These cells exhibit characteristics typical of epithelial cells and are capable of approximately 15 population doublings. Karyotyping of these cells showed that these cells are aneuploid, thereby explaining the ability of these cells to grow *in vitro* (data not shown). However, these cells go into senescense after 15 population doublings, showing that these cells are not immortalised. Furthermore, we investigated whether these cells have active Wnt signalling, the hallmark for colorectal cancer cells. A TCF reporter assay showed that there is no aberrant Wnt signalling present. Also, transient transfection of the colon epithelial cells with constructs containing wild-type or mutated Met did not lead to active Wnt signalling. This finding is in contrast to previous studies (Danilkovitch-Miagkova *et al*, 2001; Monga *et al*, 2002; Herynk *et al*, 2003) showing that activation of Met leads to activation of *β*-catenin. Although we have no formal prove for this discrepancy, we believe that this could be due to the cell system used.

Expression of WTMet or Metmut in the FHC cells lead to overexpression and to increased phosphorylation of the receptor and its effector molecules MAPK and PKB. Although the basal levels of phospho-PKB are low in colon carcinoma cells, still an activation of PKB was present after overexpression of wild-type and mutated Met. Although wild-type MET does not normally signal independent of HGF stimulation, overexpression of receptor tyrosine kinases could lead to dimerisation and autophosphorylation and hence mimicks a continuous presence of ligand (reviewed in Schlessinger, 2000). In addition to enhanced signalling of the MET pathway, E-cadherin levels were downregulated after HGF stimulation in mock- and WTMet-transfected cells and a mutated Met construct diminished E-cadherin expression in the transfected cells independent of ligand stimulation. This was shown by confocal microscopy as well as by Western blot analysis. The relative downregulation of E-cadherin, however, is underestimated due to the under-representation of transfected cells in the total pool. E-cadherin is a homotypic adhesion molecules involved in cell–cell contact (Huttenlocher *et al*, 1998; Horwitz and Parsons, 1999; Masur *et al*, 2001). Cells with a high level of E-cadherin have relatively low migratory capacity (Masur *et al*, 2001). Our results showing that overexpression of MET causes downregulation of E-cadherin expression therefore implies a higher migratory propensity of these cells. Furthermore, low E-cadherin expression levels are used as a diagnostic marker for malignant cells, indicating that the transfected colon epithelial cells are transformed. In line with these data, transfected cells containing mutant MET were able to form colonies in soft agar. This shows that these cells have higher survival and growth capacity due to increased transformation. *In vivo* experiments further confirm our hypothesis that MET plays a role in tumour formation, showing that cells overexpressing WT or mutated MET can indeed form tumours within 12 days after injection.

We show that overexpression of the receptor tyrosine kinase MET in primary colon epithelial cells activates downstream signalling molecules. Activation of this signalling pathway leads to a diminished E-cadherin expression, colony-formation capacity and subsequent tumour growth in nude mice. We propose that upregulation of MET could lead to transformation of colon epithelial cells and can initiate and enhance tumour growth in colorectal cancer irrespective of Wnt signalling.
